# The impact of different aetiologies on the cognitive performance of frontal patients

**DOI:** 10.1016/j.neuropsychologia.2014.12.025

**Published:** 2015-02

**Authors:** Lisa Cipolotti, Colm Healy, Edgar Chan, Fay Bolsover, Francesca Lecce, Mark White, Barbara Spanò, Tim Shallice, Marco Bozzali

**Affiliations:** a Department of Neuropsychology, National Hospital for Neurology and Neurosurgery, London, UK; b Dipartimento di Psicologia, Università di Palermo, Italy; c Institute of Cognitive Neuroscience, University College London, UK; d Department of Neuroradiology, National Hospital for Neurology and Neurosurgery, London, UK; e Neuroimaging Laboratory, Santa Lucia Foundation, Rome, Italy; f International School for Advanced Studies (SISSA-ISAS), Trieste, Italy

**Keywords:** Executive functions, Frontal lesions, Aetiology, Cognitive performance, Stroke, Tumour

## Abstract

Neuropsychological group study methodology is considered one of the primary methods to further understanding of the organisation of frontal ‘executive’ functions. Typically, patients with frontal lesions caused by stroke or tumours have been grouped together to obtain sufficient power. However, it has been debated whether it is methodologically appropriate to group together patients with neurological lesions of different aetiologies. Despite this debate, very few studies have directly compared the performance of patients with different neurological aetiologies on neuropsychological measures. The few that did included patients with both anterior and posterior lesions.

We present the first comprehensive retrospective comparison of the impact of lesions of different aetiologies on neuropsychological performance in a large number of patients whose lesion solely affects the frontal cortex. We investigated patients who had a cerebrovascular accident (CVA), high (HGT) or low grade (LGT) tumour, or meningioma, all at the post-operative stage. The same frontal ‘executive’ (Raven's Advanced Progressive Matrices, Stroop Colour-Word Test, Letter Fluency-S; Trail Making Test Part B) and nominal (Graded Naming Test) tasks were compared. Patients' performance was compared across aetiologies controlling for age and NART IQ scores. Assessments of focal frontal lesion location, lesion volume, global brain atrophy and non-specific white matter (WM) changes were undertaken and compared across the four aetiology.

We found no significant difference in performance between the four aetiology subgroups on the ‘frontal’ executive and nominal tasks. However, we found strong effects of premorbid IQ on all cognitive tasks and robust effects of age only on the frontal tasks. We also compared specific aetiology subgroups directly, as previously reported in the literature. Overall we found no significant differences in the performance of CVA and tumour patients, or LGT and HGT patients or LGT, HGT and meningioma's on our four frontal tests. No difference was found with respect to the location of frontal lesions, lesion volume, global brain atrophy and non-specific WM changes between the subgroups.

Our results suggest that the grouping of frontal patients caused by different aetiologies is a pragmatic, justified methodological approach that can help to further understanding of the organisation of frontal executive functions.

## Highlights

• Impact of different aetiology on frontal patient's performance.
• No difference between aetiology subgroups in frontal lesion characteristics.
• No difference between aetiology subgroups in neuropsychological performance.
• The importance of accounting for age, NART IQ and lesion characteristics.
• Grouping frontal patients by different aetiologies is methodologically appropriate.

## 1 Introduction

Neuropsychological group study methodology is considered one of the primary methods to further understanding of the neuroanatomical architecture underlying cognitive functions. However, to obtain sufficient power with this methodology it is necessary to recruit rather large numbers of neurological patients. If too few patients are used, the results are inevitably inconclusive. Thus, to investigate neuro-cognitive architectures, patients with different aetiologies such as vascular (CVA) or tumour (different types of brain tumours) are often combined. A typical example of this approach is given by research investigating the organisation of frontal ‘executive’ functions. Different aspects of executive functioning have been explored in influential studies grouping patients with frontal lesions caused by stroke or tumours. Thus, Stuss et al. (2003) investigated a measure of executive control combining patients with CVA (*n*=19) and patients with either tumour or lobectomy (*n*=12). Roca et al. (2010) investigated fluid intelligence and executive functions in a group which combined 11 CVA and 31 tumour patients. Robinson et al. (2012) investigated verbal generation in a group combining 15 CVA and 52 tumour patients.

However, it is well known that stroke and tumours affect brain structures in several different ways. For example, CVAs such as ischaemic stroke causes cell death within the affected area. In contrast, neural activity can persist in areas infiltrated by low grade tumours (e.g. Krainik et al., 2003). The onset of a CVA is defined by an acute event; the rate of brain tumour growth can vary dramatically by grading (see, e.g. Jääskeläinen et al., 1985; Kleihues et al., 2007, p. 36). Physical changes in brain structures resulting from different grades of brain tumour are not equivalent. For example, low grade tumours and meningioma's are likely to compress adjacent brain structures (Perry et al., 2007). In contrast, high grade tumours such as glioblastomas are likely to invade cortical or subcortical structures (Kleihues et al., 2007). These fundamental differences raise the possibility that CVA, high grade tumours, low grade tumours and meningioma's may recruit mechanisms of neural plasticity in different ways leading to different functional outcomes.

In the literature it has been debated if, for the purpose of neuropsychological investigation, the grouping together of patients with different neurological aetiologies is methodologically appropriate (e.g. Duffau, 2011: Clinical Neuroanatomy, discussion forum, Cortex). For example, Anderson et al. (1990) argued that as far as stroke and tumour patients are concerned “…the two patient types should be treated separately for the purpose of neuropsychological research”. Karnath and Steinbach (2011) focused on tumour patients (unilateral gliomas or meningiomas) and argued that neuropsychological investigation should not “…use patients with tumours to identify the ‘critical lesion sites’ related to a certain disorder, in particular if the more general aim is to determine the neural representation of this function in the human brain…” (p. 1005). In contrast, Shallice et al. (2010) reported that patients with different aetiologies give rise to the same localisation of a critical function (for example see Brambati et al. (2006) and Campanella et al. (2010), naming of non-living objects).

Despite this debate, very few studies have directly compared the performance of patients with different neurological aetiologies on neuropsychological measures. Only Anderson et al. (1990) have compared stroke and tumour patients and attempted to control for lesion location. The authors investigated the neuropsychological performance of a relatively small sample of stroke (*n*=19; 10 left, 9 right) and tumour (8 left, 9 right; glioma, grade unknown *n*=15; meningioma *n*=2) patients. Using mainly CT scans, the investigators attempted to match anatomically the patients for lesion size and location on a case-by-case basis. The authors reported that the left stroke patients performed significantly worse than the left tumour patients on 4/6 subtests from the Multilingual Aphasia Examination battery. For right hemisphere patients, differences were less clear cut (see Shallice et al. (2010) for discussion).

Other studies have investigated whether patients with high and low grade tumours differ in terms of neuropsychological test performance (Hom and Reitan, 1984; Hahn et al., 2003). Unfortunately, the effect of lesion location at a finer level than the hemisphere has generally not been reported. In an older study, Hom and Reitan (1984) compared patients with high grade tumours (grade≥3, *n*=46) and with low-grade tumours (grade≤2, *n*=46) on the WAIS-III and Halstead–Reitan battery. High grade performed worse than low grade tumour patients on the WAIS-III and almost all the subtests of the Halstead–Reitan battery. Similarly, Hahn et al. (2003) reported that high grade (*n*=31) performed significantly poorer than low grade tumour patients (*n*=37) on two out of ten neuropsychological measures (Trail-Making Test Part A, COWAT FAS). Shallice et al. (2010) studied the effect of type of tumour on four different ‘right parietal’ tests. They investigated high grade tumour patients (*n*=25), low grade tumour patients (*n*=28) and meningioma patients (*n*=15). The authors reported that in two of the four tests, high grade tumour patients performed significantly worse than low grade tumour patients post-operatively. However, there was a significant post-operative decline in three tests in the low grade tumour group.

In contrast, other studies have reported no significant differences between high and low grade tumour patients on extensive batteries of tests. Scheibel et al. (1996) contrasted patients with highly malignant glioblastomas (grade 4, *n*=106) and less malignant gliomas (grade≤3, *n*=139), with all patients at the post-operative stage. No effect of tumour malignancy was found, although significant effects for tumour lateralisation and type of therapy (radiotherapy, resection or both) were reported. Talacchi et al. (2011) also documented no significant difference in performance in a small number of high and low grade post-operative tumour patients (*N*=17 and *N*=12, respectively).

It should be noted that none of the studies reviewed above attempted a comprehensive comparison between aetiologies, such as vascular and different type and grades of tumours. It is often unclear whether studies reporting on tumour patients were tested at the pre-operative or post-operative stage. All previous studies have included patients with both anterior and posterior lesions. Moreover, most did not characterize lesion location at a finer level than the damaged hemisphere, the only exception being the study by Shallice et al. (2010). Lesion size comparisons have only been documented by the Anderson et al. (1990) study using mainly CT scans. Interestingly, only some of the studies have analysed and corrected for the effect of age (Hahn et al., 2003; Scheibel et al., 1996). Others have not and yet reported difference between the age of the aetiology groups (e.g. CVA patients older than tumour patients, Anderson et al., 1990; high grade glioma patients older than low grade glioma patients, Hom and Reitan, 1984).

The aim of our retrospective study was to carry out the first comprehensive comparison of the impact of different aetiologies on neuropsychological performance. We reviewed a large number of patients with CVA; high and low grade tumours as well as meningiomas, all at the post-operative stage. The lesions of all patients were unilateral and confined to the frontal lobes. We determined the location of the frontal lesions and for a subsample of patients the total lesion volume. Measures of global atrophy and white matter (WM) changes were also undertaken. We compared the performance of the frontal patients on the same frontal ‘executive’ and nominal tasks across the aetiologies whilst taking into account differences in age and premorbid levels of functioning. Apriori comparisons of specific aetiologies groups were also conducted. Using these we sought to investigate further the findings of previous studies (Anderson et al., 1990; Hom and Reitan, 1984).

## 2 Materials and methods

### 2.1 Participants

One hundred and sixty four patients with a unilateral lesion confined to the frontal lobes resulting from a cerebrovascular accident (CVA) or a brain tumour, attending the Neuropsychology Department at the National Hospital for Neurology and Neurosurgery, Queen Square, London, were retrospectively screened for study eligibility. Our exclusion criteria were (i) age>80 years, (ii) current or previous psychiatric disorders, (iii) current or previous neurological disorders including previous CVAs or tumours, (iv) presence of metastatic tumours, (v) previous chemotherapy, (vi) visual or motor impairment, (vii) previous head trauma, (viii) history of excessive alcohol or drug use, (ix) no MRI or CT scan results available, (x) no neuropsychological assessment available, (xi) a score below the 5th percentile on a test of general intelligence (WAIS-III, WAIS-R or Raven's Matrices), (xii) gross perceptual or language impairment. Non-native English speakers were only included in the study if they obtained a score at or above the 25th percentile on the National Adult Reading Test (NART, Nelson, 1982).

Application of the exclusion criteria resulted in 100 patients being retrospectively included in the study. For all patients the diagnosis was confirmed by neurological investigation. Tumour grade was confirmed by histopathological studies following resection or biopsy. All tumour patients had undergone tumour resection prior to neuropsychological assessment. Patients were assigned to four groups based on lesion aetiology; the CVA group (*n*=29), the low grade tumour group (LGT, tumour grade≤2, *n*=24), the high grade tumour group (HGT, tumour grade≥3, *n*=20) and the meningioma group (*n*=27).

Retrospective recruitment of patients was approved by the National Hospital for Neurology and Neurosurgery and the Institute of Neurology Joint Research Ethics Committee (UK). Fifty three patients with frontal lesions included in this study have had aspects of their profiles reported previously (MacPherson et al., 2008, 2010; Murphy et al., 2013; Robinson et al., 2012; Turner et al., 2007).

### 2.2 Neuroimaging analysis

Both MRI and CT data were used, as our principal goal was to recruit a large number of patients. Hard copies or digital records of all scans were reviewed by two independent neurologists (MB and BS) who were blind to the medical history of each patient. For 95 out of 100 patients MRI (*n*=76; *n*=5 hard copies) or CT scan (*n*=19) were available for analysis. Digital brain MRI scans were obtained on systems operated at .5 (*n*=1) or 1.5 (*n*=54) or 3 (*n*=16) Tesla and included the acquisition of an axial dual-echo and an axial and coronal T1-weighted scan. CT scans were obtained using spiral CT systems. Axial images were collected with an effective slice thickness of 5 mm and pitch of 1.5. Only T1-weighted MRI scans (or CT scans when MRI was not available) were used for the assessment of frontal lesions. We conducted an analysis of the total frontal lesion volumes only for a subset of patients. These were the patients for whom we obtained the largest number of MRI scans at the same magnetic strength (1.5 T). DE and FLAIR images were used for the assessment of global brain atrophy and non-specific WM changes.

#### 2.2.1 Investigation of the frontal lesions

The exclusion criteria and lesion assessment guidelines were rigorous and based on detailed anatomical localisation using standard atlases (Duvernoy et al., 1991). Of note, all frontal lesions had to be entirely located within the frontal lobe. The lesion localisation method is described in detail in Robinson et al. (2012) and Murphy et al. (2013). Briefly, each frontal patient was coded for the presence of lesion and oedema in each hemisphere in the anterior and posterior portion of nine left and nine right frontal subregions (18 subregions in total). A subregion was only coded as damaged if at least 25% was affected. To compare whether left or right frontal lobe lesion differentially impact on cognitive performance across the four aetiologies, we merged the nine left and right brain subregions, and divided the patients into two groups: left and right frontal according to which hemisphere was damaged.

To investigate whether across the four aetiologies there were differences in the number of patients with lesions in different areas of the frontal lobe we employed the grouping method described by Stuss et al. (1998), Stuss and Alexander (2005) and adopted in our previous studies (e.g. Murphy et al., 2013; MacPherson et al., 2010; Robinson et al., 2012). Lesions in prefrontal subregions were grouped together to define a primary lesion site of one of four main subgroups: medial, left lateral, right lateral and orbitofrontal. For these four areas the primary lesion site was defined as either (a) damage restricted to the cortical subregions that defined the area or (b) damage affecting at least three cortical subregions used to define each area and no more than one other subregion (secondary site) belonging to an adjacent area. According to this criterion, one frontal patient only was excluded from this analysis as the criteria for the primary lesion site fit more than one subgroup. Patients with unilateral primary damage to the medial area had lesions affecting the left/right cingulate gyrus (anterior/posterior), the left/right sub-genu, and the left/right medial and superior frontal gyrus (anterior/posterior). These frontal subregions correspond to the following Brodmann areas: 6, 8, 9, 10, 23, 24, 32 and 33. Patients with damage to the lateral areas (left or right) had lesions affecting the left or right lateral part of the superior frontal gyrus (anterior/posterior), the left or right middle frontal gyrus (anterior/posterior), and the left or right inferior frontal gyrus (anterior/posterior). These subregions correspond to the Brodmann areas: 6, 8, 9, 38, 44, 45, 46 and 47. Finally, patients with brain damage to the orbitofrontal area had lesions affecting the left and right orbital cortex (Brodmann areas 10 and 11).

#### 2.2.2 Volumetric investigation of frontal lesions

A volumetric lesion analysis was conducted on the subsample of 54 patients who had a digital 1.5 T MRI scan. Two of these patients were removed due to movement artefacts. In all patients hypointense lesions were outlined on the T1-weighted MRI slices using a semi-automated local thresholding contouring software (Jim 5.0, Xinapse System, Leicester, UK, http://www.xinapse.com/). The total lesion volume was then calculated for every patient.

#### 2.2.3 Investigation of brain atrophy

Visual quantification of global brain atrophy was assessed using FLAIR or CT scans, according to the method proposed by Scheltens et al. (1997). Scores ranged from 0=absence of atrophy; 1=minimal atrophy; 2=moderate atrophy; to 3=severe atrophy. Intra- and inter-observer variability in quantifying atrophy was tested and no significant differences were observed.

#### 2.2.4 Investigation of white matter changes

To visually quantify WM changes we used the Fazekas' rating scale (Fazekas et al., 1987), which is widely used in the literature. It includes 2 sub-scales; periventricular WM changes (i.e. PVH sub-scale) and deep WM changes (i.e. DWMH sub-scale). For MRI scans, WM changes are defined as hyper-intense areas, detectable on DE and/or FLAIR images. For CT scans, WM changes are defined as hypo-dense areas within the WM. Scores range from 0 – absence of WM changes to 3 – most severe degree of WM changes.

### 2.3 Neuropsychological investigation

The neuropsychological battery comprised a series of widely used clinical tests with published standardised normative data collected from large control samples. The National Adult Reading Test (NART) was given to estimate optimal pre-morbid levels of functioning (Nelson, 1982). The results from four further neuropsychological tests were examined:

1. Raven's Advanced Progressive Matrices (RAPM; Raven, 1965), an untimed, relatively culture-free, non-verbal test of abstract reasoning, requiring the selection of the missing piece from a pattern. The total number of correct responses in Set I was recorded.
2. Stroop Test (Trenerry et al., 1989), a test of response inhibition. The total number of colour words for which the colour was correctly named in two minutes was recorded.
3. Letter fluency ‘S’ Test (FAS; Benton, 1968), a test of phonemic fluency, requiring the oral generation of words beginning with the letter S. The total number of correct responses in one minute was recorded.
4. Trail Making Test, Part B (Reitan and Wolfson, 1985), a test of task switching. The total completion time in seconds was recorded.
5. The Graded Naming Test (GNT; McKenna and Warrington 1980), a test of object naming. The total number of objects correctly named was recorded.

### 2.4 Statistical analysis

All statistics were carried out using SPSS Statistics (http://www-01.ibm.com/software/analytics/spss/).

#### 2.4.1 Demographic variable analysis

The demographic variables were analysed using chi-square analysis (handedness, gender) or an analysis of variance (ANOVA) (age, NART and years of education). This was used to test for significant difference between the four aetiologies (CVA, HGT, LGT, and meningioma).

#### 2.4.2 Neuroimaging statistical analysis

Chi-square analysis (frontal lesions), one-way ANOVA (frontal lesions volume) and Kruskal–Wallis non-parametric ANOVAs (brain atrophy, WM changes) were used in the neuroimaging analysis to test for significant differences between the aetiology subgroups. There was a significant difference in variance between aetiology subgroups in frontal lesion volumes. This was corrected with a log_10_ transformation. To account for the two sub-scales used in the WM analysis, Kruskal–Wallis non-parametric ANOVA was corrected using a Bonferroni adjustment (*p*<.025). Brain atrophy and WM changes are known to be associated with aging (e.g. Kim et al., 2011; de Leeuw et al., 2001). To examine the impact of age on brain atrophy and WM changes, linear regression analyses were conducted on brain atrophy and WM rating scale using age as predictor variable.

#### 2.4.3 Neuropsychological statistical analysis

The neuropsychological findings were analysed to ascertain if they were normally distributed and to identify outliers. The findings were also assessed for homogeneity of variance. Negative skew was found for the GNT and the Stroop Test. This was corrected using square root transformation (the square root of, the maximum possible score plus one, minus each patient′s score). A positive skew was found for Trail Making Part B. This was corrected with a log_10_ transformation. All neuropsychological data was analysed using ANCOVAs, with age and NART as covariates. Partial eta squared was used to estimate the effects size for age, NART IQ and aetiology. To investigate the effect of lateralisation, patients were classified according to hemisphere damage (left frontal lesion or right frontal lesion) and their neuropsychological performance was examined. To investigate the effect of aetiology, patients were classified according to their aetiology subgroups (CVA, LGT, HGT and Meningioma) and their neuropsychological performance was examined.

To examine the impact of the covariates on performance, linear regression analysis was conducted on each of the frontal tasks using age and NART as predictor variables. Additionally three planned comparisons of specific aetiology subgroups, motivated by previous studies, were carried out. Individual ANCOVAs were used to compare the neuropsychological performance of (i) CVA and one group including all tumour patients (LGT, HGT and meningiomas combined; Anderson et al., 1990), (ii) LGT and HGT (Hom and Reitan, 1984) and (iii) different tumour aetiologies (LGT, HGT and meningioma patients). Age and NART IQ scores were used as covariates in all analyses.

## 3 Results

### 3.1 Demographic results

Demographic results for the four aetiology subgroups (CVA, LGT, HGT and Meningioma) are shown in Table 1. No significant difference was found between patients for handedness, gender and education. There was a significant main effect of age (*F*(3, 96)=10.581, *p*<.001). Post hoc analysis revealed that the Meningioma group were significantly older than all other aetiology subgroups (CVA, *p*=.009; LGT, *p*<.001; HGT, *p*=.006) and there was a trend for LGT to be somewhat younger then CVA *(p*=.09). There was no significant main effect of NART IQ (*F*(3, 91)=1.882, *p*>.138).

### 3.2 Investigation of frontal lesions

**Fig. 1:**
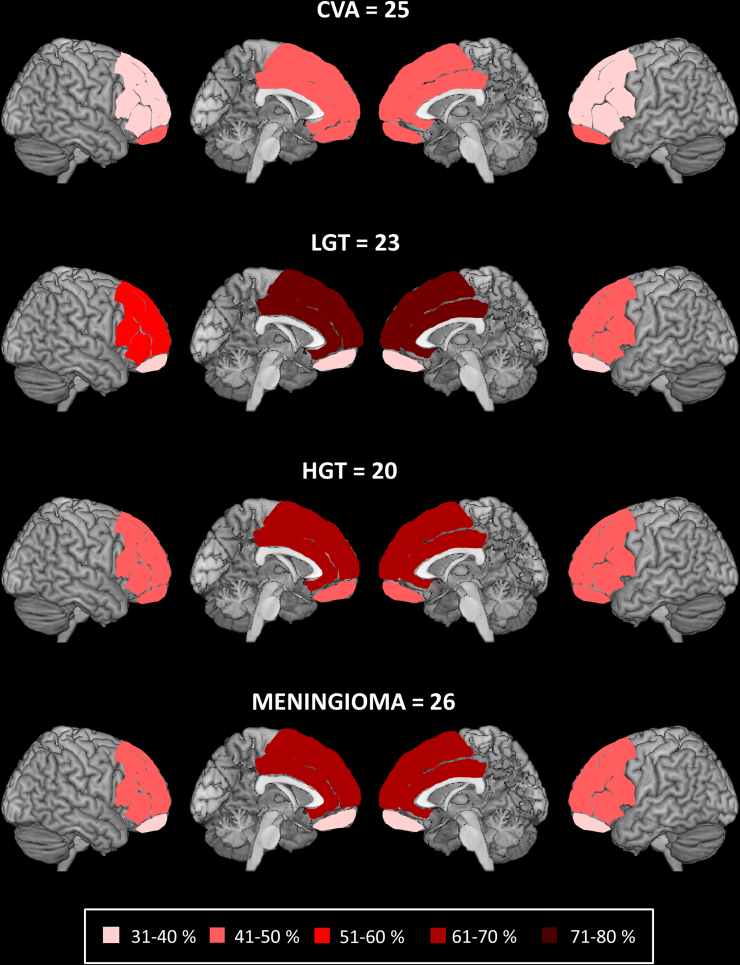
Percentage of patients with damage to the different frontal areas in each aetiology subgroup. The number of patients in each aetiology subgroup for whom we analysed frontal lesion location. Shading illustrates the percentage of patients with damage to lateral (right and left), medial and orbito-frontal regions for each aetiology subgroup. *Abbreviations*: CVA=stroke; LGT=low grade tumour; HGT=high grade tumour.

T1-w MRI or CT scans were available for 94 out of the 95 patients. We compared the four aetiology subgroups on the number of patients with focal brain damage in the four frontal areas. There was no difference across the aetiology subgroups in the numbers of patients with damage to medial (*χ*^2^(3, *N*=94)=5.929, *p*=.115), left lateral (*χ*^2^(3, *N*=94)=1.569, *p*=.666), right lateral: (*χ*^2^(3, *N*=94)=1.308, *p*=.727) and orbital frontal areas: (*χ*^2^(3, *N*=94)=1.097, *p*=.778; see Fig. 1).

### 3.3 Volumetric investigation

Volumetric analysis was available for 52 out of 95 patients (CVA=13, LGT=16, HGT=10 and meningioma=13). We found no significant difference in the total frontal lesion volume between the four aetiology subgroups *F*(3, 48)=1.384, *p*=.259.

### 3.4 Investigation of brain atrophy

FLAIR or CT scans were available for 80 out of the 95 patients. These were analysed using Scheltens et al. (1997) method of global brain atrophy quantification. A Kruskal–Wallis non-parametric ANOVA indicated that there was no significant difference across the four aetiology subgroups (*χ*^2^(3, *N*=80)=4.633, *p*=.201). This suggests that the amount of brain atrophy did not differ between patients from different aetiology subgroups. A linear regression analysis indicated that there was a significant effect of age on the increment scores on the brain atrophy rating scale (*R*^2^=.27, *F*(1, 87)=32.13, *p*<.001).

### 3.5 Investigation of white matter changes

DE, FLAIR or CT scans were available for 80 out of the 95 patients. These were analysed using the Fazekas' rating scale for the quantification of WM changes. Table 2 displays the means and standard deviations of the scores of four aetiology subgroups on the Fazekas sub-scales. Despite the rather high variability in the scores, we found a significant difference between the four aetiology subgroups in both periventricular hyper/hypo intensity (PVH) and deep white matter hyper/hypo intensity signal (DWMH) sub-scales (PVH: *χ*^2^ (3, *N*=80)=13.843, *p*=.003; DWMH: *χ*^2^ (3, *N*=80)=12.392, *p*=.006). Post-hoc pair-wise comparisons using Mann–Whitney non parametric analysis indicated that these differences were due to meningioma patients having more severe white matter changes than the others patient subgroups (**PVH**: LGT vs CVA: z=9.598, *p*=.150; LGT vs HGT: *z*=–14.003, *p*=.055; LGT vs meningioma: *z*=−24.566, *p*<.001; CVA vs HGT: *z*=−4.405, p=.529; CVA vs meningioma: *z*=−14.967, *p*=.020; HGT vs meningioma: *z*=−10.562, *p*=.135; **DWMH**: LGT vs HGT: z=−.770, p=.895; LGT vs CVA: z=2.677, p=.615; LGT vs meningioma: *z*=−16.258, *p*=.003; HGT vs CVA: *z*=1.908, *p*=.733; HGT vs meningioma: *z*=−15.489, *p*=.006; CVA vs meningioma: *z*=−13.581, *p*=.008).

Linear regression analyses indicated that there was a significant effect of age on the increment scores on both Fazekas subscales (PVH: *R*^2^=.17, *F*(1, 78)=16.37, *p*<.001; DWMH: *R*^2^=.17, *F*(1, 78)=16.09, *p*<.001). As stated above, the patients in the meningioma group were significantly older than the other three groups. To test if the severity of WM changes can be attributed to differences in age we calculated a decrement score for each patient. A decrement score was defined as the patients’ observed white matter score on each Fazekas scales minus their predicted white matter scores based on the results of the linear regression analyses. The decrement scores were then used to compare the white matter changes between the four aetiologies subgroups using Kruskal–Wallis non parametric ANOVA. After accounting for age, no significant difference was found between the four aetiologies in both periventricular hyper/hypo intensity (PVH) and deep white matter hyper/hypo intensity signal (DWMH) sub-scales (PVH: *χ*^2^ (3, *N*=80)=4.267, *p*=.234; DWMH: *χ*^2^ (3, *N*=80)=2.580, *p*=.461). This indicated that differences between the aetiology subgroups in the severity of WM changes can be attributed to differences in the ages of the aetiology subgroups.

### 3.6 Neuropsychological performance of left and right frontal patients

We compared the performance of left and right frontal patients on the neuropsychological tests. Age and NART IQ significantly affected performance on RAPM (*F*(1, 66)=14.018, *p*<.001, *η_p_*^2^=.175 and *F*(1, 66)=10.203, *p*=.002, *η_p_*^2^=.134, respectively), Stroop Test (*F*(1, 53)=57.464, *p*<.001, *η_p_*^2^=.520 and *F*(1, 53)=7.473, *p*=.008, *η_p_*^2^=.124, respectively) and Letter Fluency ‘S’ Test (*F*(1, 82)=14.630, *p*<.001, *η_p_*^2^=.151 and *F*(1,82)=12.674, *p*=.001, *η_p_*^2^=.134, respectively). There was a significant effect of age on Trail Making Test Part B (*F*(1, 58)=34.298, *p*<.001, *η_p_*^2^=.372). There was a significant effect of NART IQ on GNT score (*F*(1, 87)=83.484, *p*<.001, *η_p_*^2^=.490).

We found no significant main effect of group (left frontal vs right frontal) for the RAPM (*F*(1, 66)=.043, *p*=.837, *η_p_*^2^=.001), Letter Fluency ‘S’ Test (*F*(1, 82)=1.328, *p*=.252, *η_p_*^2^=.016), Trail Making Test Part B (*F*(1, 58)=1.84, *p*=.18, *η_p_*^2^=.031) and the GNT (*F*(1, 87)=.734, *p*=.394, *η_p_*^2^=.008). However, a significant group effect was found on the Stroop Test (*F*(1, 53)=13.348, *p*=.001, *η_p_*^2^=.201), reflecting worse performance of the left frontal patients than right frontal patients (see Table 3), as often reported (for a meta-analysis see Demakis (2004)).

### 3.7 Neuropsychological results: Performance of patients with different aetiologies

We compared the performance of the four different aetiology subgroups (CVA, LGT, HGT and Meningioma) on the five neuropsychological tests (RAPM, Stroop Test, Letter Fluency ‘S’ Test, Trail Making Test Part B and GNT).

**Fig. 2:**
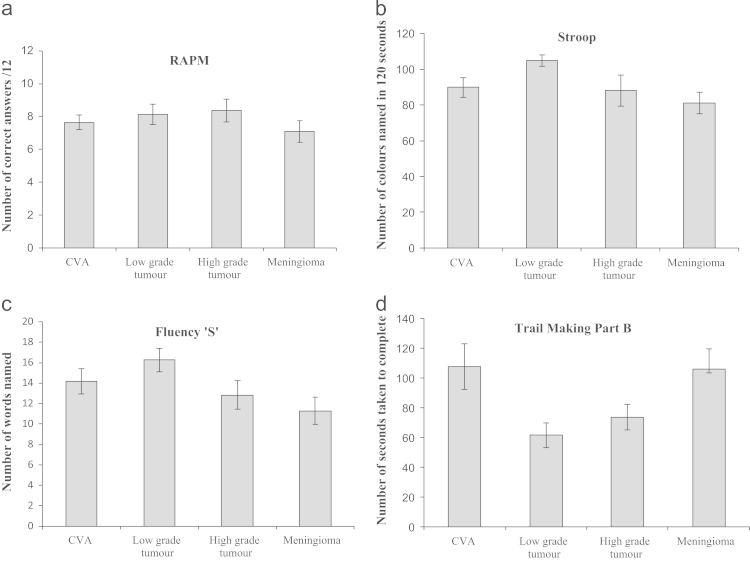
Performance of on the four frontal tasks for each of the four different aetiologies. Mean scores of the four aetiology subgroups on the four frontal tests not accounting for age and NART IQ: (a) Raven's Advanced Progressive Matrices, (b) Stroop Colour Word Task, (c) Letter Fluency ’S’, (d) Trail Making Test Part B. Error Bars represent ±1 standard error.

***RAPM***. There were significant effects of age and NART IQ on patients' performance on the RAPM (*F*(1, 64)=13.328, *p*=.001, *η_p_*^2^=.172 and *F*(1, 64)=14.177, *p*<.001, *η_p_*^2^=.181, respectively). However, there was no main effect of group, with no significant difference in the neuropsychological performance of the four aetiology subgroups (*F*(3, 64)=1.578, *p*=.203, *η_p_*^2^=.069, see Fig. 2a).

***Stroop Test**.* Age and NART IQ significantly affected performance (*F*(1, 51)=31.623, *p*<.001, *η_p_*^2^=.383 and *F*(1, 51)=6.541, *p*=.014, *η_p_*^2^=.114, respectively). Again we found no main effect of group, with no significant difference in performance between the four aetiology subgroups (*F*(3, 51)=.199, *p*=.896, *η_p_*^2^=.012, see Fig. 2b).

***Letter Fluency ‘S’ Test***. Age and NART IQ significantly affected performance (*F*(1, 80)=8.078, *p*=.006, *η_p_*^2^=.092 and *F*(1, 80)=11.378, *p*=.001, *η_p_*^2^=.125, respectively). Again there was no main effect of group, with no significant difference in performance between the four aetiology subgroups (*F*(3, 80)=.319, *p*=.812, *η_p_*^2^=.012, see Fig. 2c).

***Trail Making Test Part B**.* Age significantly affected performance (*F*(1, 56)=21.767, *p*<.001, *η_p_*^2^=.280). NART IQ trended towards significantly affecting performance (*F*(1, 56)=3.506, *p*=.066, *η_p_*^2^=.059). There was no significant difference in performance on this task between the four aetiologies after accounting for age and NART IQ scores (*F*(3, 56)=1.796, *p*=.158, *η_p_*^2^=.088, see Fig. 2d).

***Graded Naming Test.*** Only NART IQ was found to significantly affect performance (*F*(1, 85)=82.171, *p*<.001, *η_p_*^2^=.492). There was no main effect of group, with no significant difference in performance between the four aetiology subgroups (*F*(3, 85)=2.02, *p*=.117, *η_p_*^2^=.067).

#### 3.7.1 Cognitive performance of the patients’ subsample within volumetric analysis

We also compared the cognitive performance of the subsample of 52 patients for whom we conducted a volumetric lesion analysis. Similarly to the results reported above, we found no significant difference across the four different aetiology groups on the five neuropsychological tests (RAPM: *F*(3, 29)=.390, *p*=.761, *η_p_*^2^=.039; Stroop Colour-Word test: *F*(3, 26)=.855, *p*=.477, *η_p_*^2^=.09; Letter Fluency ‘S’: *F*(3, 38)=.447, *p*=.721, *η_p_*^2^=.034; Trails Making test Part B: *F*(3, 28)=2.226, *p*=.107, *η_p_*^2^=.193; GNT: *F*(3, 41)=.526, *p*=.667, *η_p_*^2^=.037).

### 3.8 Investigation of the effects of age and premorbid IQ on cognitive performance

The previous analyses demonstrated that age and NART IQ have a significant effect on patients' performance. To further examine this, linear regression analyses were performed on all tests, using age and NART IQ as predictor variables. The results indicated that age and NART IQ combined significantly predicted performance on all of the frontal executive tests. Furthermore, age significantly accounted for variance in the four frontal executive tests but not in the GNT, a test of nominal function. NART IQ was a significant predictor of performance on all cognitive tests (see Table 4).

### 3.9 Specific aetiology subgroups analysis

We conducted three more specific analyses comparing aetiologies. Two of these analyses were used to compare our data with previous studies (Anderson et al., 1990; Hom and Reitan, 1984).

#### 3.9.1 Performance of CVA and tumour (LGT, HGT and meningioma) patients

In accordance with Anderson et al. (1990), the performance of the CVA patients was compared with a combined group of all tumour patients (LGT, HGT and meningioma). Laterality of lesion was added as an additional independent variable since Anderson et al. (1990) reported that following left hemisphere damage stroke patients performed significantly poorer than tumour patients on selected subtests of the Multilingual Aphasia Examination Battery. We found a significant effect of age and NART IQ on the RAPM (*F*(1, 64)=14.366, *p*<.001, *η_p_*^2^=.183 and *F*(1, 64)=9.007, *p*=.004, *η_p_*^2^=.123, respectively) Stroop Colour-Word Test (*F*(1, 51)=56.404, *p*<.001, *η_p_*^2^=.525 and *F*(1, 51)=7.825, *p*=.007, *η_p_*^2^=.133, respectively), Letter Fluency ‘S’ Test (*F*(1, 80)=13.907, *p*<.001, *η_p_*^2^=.148 and *F*(1, 80)=12.245, *p*=.001, *η_p_*^2^=.133, respectively). There was a significant effect of age on performance on Trail Making Test Part B (*F*(1, 56)=35.586, *p*<.001, *η_p_*^2^=.389). On the GNT, there was a significant effect of NART IQ on performance (*F*(1, 85)=81.975, *p*<.001, *η_p_*^2^=.491).

We found no main effect of group, with no significant difference in performance between CVA and tumour aetiology subgroups on the RAPM (*F*(1, 64)=.138, *p*=.711, *η_p_*^2^=.002); Stroop Colour-Word Test (*F*(1, 51)=.450, *p*=.506, *η_p_*^2^=.009); and Letter Fluency ‘S’ Test (*F*(1, 80)=.226, *p*=.636, *η_p_*^2^=.003). We found a significant difference in the performance of CVA and tumour patients on the Trail Making Test Part B (*F*(1, 56)=4.890, *p*=.031, *η_p_*^2^=.08) and a non-significant trend on the GNT (*F*(1, 85)=3.202, *p*=.077, *η_p_*^2^=.036). Given these two latter results we conducted post-hoc analyses for the Trail Making Part B and GNT tests comparing the performance of the CVA patients with each tumour subgroup. We found no significant difference in Trail Making part B and GNT tests' performance between CVA and LGT (Trail Making Test Part B, *F*(1, 31)=2.443, *p*=.128, *η_p_*^2^=.073; GNT (*F*(1, 43)=2.808, *p*=.101, *η_p_*^2^=.061). Similarly we found no significant difference between CVA and meningioma’s in performance on the Trail making test B and on the GNT (*F*(1, 29)=2.203, *p*=.149, *η_p_*^2^=.071 and *F*(1, 46)=.369, *p*=.547, *η_p_*^2^=.008). However we found a significant difference between CVA and HGT in performance on the GNT (*F*(1, 40)=5.335, *p*=.026, *η_p_*^2^=.118) and a non-significant trend on the Trail Making Test Part B (*F*(1, 30)=3.223, *p*=.083, *η_p_*^2^=.097; suggesting that the CVA patients performed worse than HGT on these two tests.

We found no main effect of laterality, with no difference in the performance of left and right frontal patients on the RAPM (*F*(1, 64)=.230, *p*=.633, *η_p_*^2^=.004), Letter Fluency ‘S’ Test (*F*(1, 80)=.731, *p*=.395, *η_p_*^2^=.009), Trail Making Test Part B (*F*(1, 56)=.804, *p*=.374, *η_p_*^2^=.014), the GNT (*F*(1, 85)=.200, *p*=.656, *η_p_*^2^=.002). The only exception was the Stroop Colour-Word Test (*F*(1, 51)=13.654, *p*=.001, *η_p_*^2^=.211) where left frontal patients performed worse than right frontal patients.

We found no significant interactions between aetiology and laterality on any neuropsychological tasks (RAPM: *F*(1, 64)=.449, *p*=.505, *η_p_*^2^=.007; Stroop Colour-Word Test: *F*(1, 51)=.741, *p*=.393, *η_p_*^2^=.014; Letter Fluency ‘S’ Test: *F*(1,80)=.048, *p*=.826, *η_p_*^2^=.001; Trail Making Test Part B: *F*(1, 56)=1.477, *p*=.229, *η_p_*^2^=.026; and GNT: *F*(1, 85)=.129, *p*=.720, *η_p_*^2^=.002).

#### 3.9.2 Performance of LGT and HGT patients

The performance of the LGT and HGT patients was compared (Hom and Reitan, 1984). There was a significant effect of NART IQ on performance on the RAPM (*F*(1, 24)=5.95, *p*=.022, *η_p_*^2^=.199), Stroop Colour-Word Test (*F*(1, 23)=4.839, *p*=.038, *η_p_*^2^=.174), Letter Fluency ‘S’ Test (*F*(1, 36)=8.526, *p*=.006, *η_p_*^2^=.191), Trail Making Test Part B (*F*(1, 25)=10.06, *p*=.004, *η_p_*^2^=.287) and GNT (*F*(1, 37)=24.867, *p*<.001, *η_p_*^2^=.402). For all neuropsychological tests, there was no main effect of group, with no significant difference in performance between LGT and HGT aetiology subgroups: Stroop Colour-Word Test (*F*(1, 23)=.480, *p*=.495, *η_p_*^2^=.02), Letter Fluency ‘S’ Test (*F*(1,36)=.38, *p*=.542, *η_p_*^2^=.01), Trail Making Test Part B (*F*(1, 25)=.246, *p*=.624, *η_p_*^2^=.01), GNT (*F*(1, 37)=.516, *p*=.477, *η_p_*^2^=.014). However there was a trend towards significance for the RAPM (*F*(1, 24)=3.318, *p*=.081, *η_p_*^2^=.121).

#### 3.9.3 Performance of LGT, HGT and meningioma patients

The performance of the LGT, HGT and Meningioma patients was compared. There was a significant effect of age and NART IQ on performance on the RAPM (*F*(1, 45)=6.204, *p*=.017, *η_p_*^2^=.121 and *F*(1, 45)=12.945, *p*=.001, *η_p_*^2^=.223, respectively), the Stroop Colour-Word Test (*F*(1, 34)=9.378, *p*=.004, *η_p_*^2^=.216 and *F*(1, 34)=8.849, *p*=.005, *η_p_*^2^=.207, respectively), Letter Fluency ‘S’ Test (*F*(1, 57)=5.778, *p*=.019, *η_p_*^2^=.092 and *F*(1, 57)=10.682, *p*=.002, *η_p_*^2^=.158, respectively) and Trail Making Test Part B (*F*(1, 37)=7.510, *p*=.009, *η_p_*^2^=.169 and *F*(1, 37)=5.716, *p*=.022, *η_p_*^2^=.134, respectively). On the GNT, there was only a significant effects of NART IQ on performance (*F*(1, 61)=58.917, *p*<.001, *η_p_*^2^=.491). For all neuropsychological tests, there was no main effect of group, with no significant difference in performance between the three aetiology subgroups on the RAPM (*F*(2, 45)=2.224, *p*=.120, *η_p_*^2^=.09), Stroop Colour-Word Test (*F*(2, 34)=.182, *p*=.835, *η_p_*^2^=.011), Letter Fluency ‘S’ Test (*F*(2, 57)=.168, *p*=.845, *η_p_*^2^=.006), Trail Making Test Part B (*F*(2, 37)=.142, *p*=.868, *η_p_*^2^=.008) and GNT (*F*(2, 61)=.849, *p*=.433, *η_p_*^2^=.027).

## 4 Discussion

To the best of our knowledge, our retrospective study of a large sample of frontal patients is the first to compare cognitive performance across four different aetiologies. The frontal patients' lesions were confined to the left or right frontal lobe and performance was assessed with four frontal executive tasks and one nominal task. Thus, our comparison of cognitive performance is less affected by variability in lesion location than earlier studies. Our four aetiology subgroups (CVA, high or low grade tumour and meningioma) did not differ in handedness, gender, education or premorbid intelligence. There was a significant effect of age, with LGT patients being somewhat younger and meningioma patients significantly older. This is in keeping with the literature reporting that LGT generally affects younger adults (mean age of occurrence of 35 years; e.g. Behin et al. (2003)), whilst meningiomas generally affect older adults (mean age of diagnosis of 56 years; e.g. Milker-Zabel and Debus, (2008).

We found no significant difference across the four aetiologies in the number of patients with damage to lateral (left and right), medial and orbitofrontal regions. Similarly, no significant difference across the four aetiologies was found in the total frontal lesion volumes or the amount of global brain atrophy. We did find significantly more white matter changes in the meningioma group. However, when we compared white matter changes, after accounting for age, no significant difference between the aetiology subgroups was found. This suggests that the reported difference in white matter changes is most likely due to age-related changes. Indeed it is known that age is associated with an increase in white matter changes (e.g. Xiong and Mok, 2011).

We found that the four aetiology subgroups did not significantly differ in their performance on four ‘frontal’ executive test (RAPM, Stroop Test, Trail Making Test Part B, Letter Fluency ‘S’) and on a nominal test (GNT). A previous study on a rather small sample of patients with frontal lobe epilepsy similarly reported no effect of aetiology on frontal tasks (Upton and Thompson, 1997). Of course, in considering non-significant results one always has the issue of statistical power. Our group size of 100 frontal patients is larger than that of the three typical ‘frontal’ studies quoted in the introduction (Stuss et al., 2003; Roca et al., 2010; Robinson et al., 2012). Furthermore, the effect sizes we reported suggest that a vastly larger patient sample would be needed to observe any potential differences in the neuropsychological performance between aetiologies. For example, based a power of .8, *α*=.05 and on the effect size obtained in the current study, 902 patients would have been needed to achieve a significant effect of aetiology on the Stroop task (using the method prescribed by Faul et al. (2007)).

Interestingly, while not finding an effect of aetiology on cognitive performance, we did document a significant effect of premorbid IQ on all cognitive tasks and a significant effect of age on the four frontal executive tests. In keeping with our current findings, a decline in performance on frontal ‘executive’ tasks has been reported in healthy aging and in patients with head injury (Ardila and Rosselli, 1989; Whelihan and Lesher, 1985; Raymont et al., 2008; Senathi-Raja et al., 2010). This supports the hypothesis that cognitive processes supported by the frontal lobes are among the first to decline with age (Albert and Kaplan, 1980; Daigneault et al., 1992; Dempster, 1992). Thus when comparing across aetiologies it is important to account for age because of the known differences between the aetiologies in the age of onset (for example see Behin et al. (2003)).

We conducted three more focused analyses comparing specific aetiology subgroups directly. These comparisons have been previously carried out by others, although no previous studies compared all four aetiologies, with all tumours at the post-operative stage. We first compared CVA and tumours. Again, no significant difference between aetiologies was found in performance on all but one of the neuropsychological test. The only exception was found on Trail-Making Test part B; however, this result would not survive Bonferroni correction since there is no a priori reason to assume that aetiology should affect only this particular executive test. The lack of difference in performance across CVA and tumours is in contrast with the only study which directly compared these two aetiologies (Anderson et al., 1990). Notably, Anderson and colleagues’ did not control for the effect of age, even though their CVA patients were older than their tumour patients. This raises the possibility that their reported differences on neuropsychological test performance may be due to the older age of CVAs, rather than to differences in aetiology. When the effect of age is controlled for, as in our study, the performance of CVA and Tumour patients tended not to show significant effects.

Secondly, we compared the neuropsychological performance of the LGT and HGT groups. We found no significant difference in cognitive performance between the tumour types. The only exception was a non-significant trend with LGT tending to perform worse on the RAPM. Two previous studies also reported no significant difference in performance between LGT and HGT patients, at the post-operative stage of treatment (Scheibel et al., 1996; Talacchi et al., 2011). In other studies, complex patterns of aetiology effects have been reported with usually poorer cognitive performance associated with HGT (e.g. Shallice et al., 2010; Talacchi et al., 2011). It is possible that some but not all of the previously reported differences in performance between high and low grade tumours may have been due to patients being at the preoperative or postoperative stage rather than to different tumour types (e.g. Hahn et al., 2003; Hom and Reitan, 1984; but see Shallice et al. (2010)). Pre-operatively, low and high grade tumours may recruit mechanisms of neural plasticity in different ways, leading to different functional outcomes. For example, functional networks in low grade but not high grade tumours have been shown to differ from healthy controls, possibly reflecting differences in plasticity when compensating for different lesion growth patterns (Van Dellen et al., 2012).

In the literature it has been debated whether the grouping of patients with different neurological aetiologies is methodologically appropriate for the purpose of neuropsychological investigation. When investigating neuro-cognitive architecture it is critical to have sizeable groups of patients so sufficient power can be obtained to search for significant effects. Our main result indicated that after accounting for the significant effects of age and premorbid IQ on cognitive performance there were no significant differences between frontal patients of different aetiologies. This result suggests that the grouping together of patients with different aetiologies but similar site and size of lesions is a pragmatic procedure that helps to make this possible. In our view any averaging procedure of neuropsychological patients is liable to suffer from potential artefacts, but to eliminate all possible artefacts makes it impossible in practise to obtain useful results. One must balance the danger of artefacts with the utility of increased sample size. Our study has established that combining across vascular and different types of tumour pathologies is not likely to produce a major distortion in the pattern of neuropsychological performance, at least for frontal patients. Our results therefore suggest that grouping patients with focal frontal lesions caused by different aetiologies is a pragmatically justified methodological approach that can help to further the understanding of the organisation of frontal executive functions.

## Figures and Tables

**Table 1:** Demographic results.

**Groups**	**Handedness (L/R)**	**Gender (M/F)**	**Age (years) mean (SD)**	**NART IQ mean (SD)**	**Education (years) mean (SD)**
**CVA (*n***=**29)**	4/25	16/13	46.59 (17.71)	107.15 (9.99)	14.07 (3.46)
**LGT (*n***=**24)**	3/21	14/10	37.78 (7.08)	112.30 (9.17)	14.25 (2.92)
**HGT (*n***=**20)**	2/18	13/7	44.95 (14.51)	104.16 (14.31)	14.3 (2.7)
**Meningioma(n=27)**	4/23	11/16	58.19 (10.70)	108.48 (11.95)	13.78 (3.21)

CVA=stroke, LGT=low grade tumour, HGT=high grade tumour, L=left, R=right, M=male, F=female, N=number, SD=standard deviation.

**Table 2:** Mean and standard deviation of the aetiology subgroups on the severity of white matter change.

**Aetiology**	**Fazekas' WM changes rating sub-scale**	
	**PVH mean**	**DWMH mean**
**CVA (SD)**	.65 (.78)	.17 (.39)
**LGT (SD)**	.32 (.58)	.11 (.32)
**HGT (SD)**	.75 (.58)	.13 (.34)
**Meningioma (SD)**	1.23 (.87)	.68 (.89)

WM=white matter; PVH=periventricular hyper intensity scale; DWMH=deep white matter hyper intense/hypo dense signal scale; CVA=stroke; LGT=low grade tumour; HGT=high grade tumour.

**Table 3:** Neuropsychological results: left and right frontal patients.

**Measure**	**LF (*N***=**44)**	**RF (*N***=**56)**
**NART Mean Full Scale IQ (SD)**	107.48 (13.58)	108.74 (9.68)
**RAPM No Correct/12 (SD)**	7.94 (2.60)	8.27 (2.72)
**STROOP No of colours in 120 s (SD)**	77.81 (26.91)	95.74 (22.92)
**FLUENCY ‘S’ No of words in 60 s (SD)**	9.78 (5.99)	13.55 (4.60)
**Trails B No of seconds (SD)**	96.21 (38.14)	91.61 (72.29)
**GNT No Correct/30 (SD)**	18.72 (4.68)	21.50 (4.48)

LF=left frontal, RF=right frontal, No=number, NART=National Adult Reading Test, RAPM=Raven's Advanced Progressive Matrices, GNT=Graded Naming Test, N=number of participants, s=seconds and SD=standard deviation presented in parentheses.

**Table 4:** Regression analyses for each neuropsychological test with age and NART IQ as predictor variables.

**Test**	**Model**		**Age**		**NART**	
	***R***^**2**^	***P*-value**	**Beta (SE)**	***P*-value**	**Beta (SE)**	***P*-value**
**RAPM**	.265	<.001	−.067 (.018)	<.001	.072 (.022)	.002
**Stroop**	.488	<.001	.126 (.018)	<.001	−.063 (.023)	.008
**Fluency ‘S’**	.242	<.001	−.149 (.039)	<.001	.191 (.052)	<.001
**Trail B**	.390	<.001	.009 (.001)	<.001	−.004 (.002)	.044
**GNT**	.489	<.001	−.003 (.004)	.427	−.047 (.005)	<.001

NART=National Adult Reading Test; RAPM=Raven's Advanced Progressive Matrices; GNT=Graded Naming Test; ±1 standard error are presented in parentheses; *R*2=variance accounted for when age and NART are both used as predictors.
